# Thymol, Alpha‐Tocopherol and Ascorbyl Palmitate as a Natural Feed Supplements to Modulate Immune Variables and Heterophil to Lymphocyte Ratio in Broiler Chickens

**DOI:** 10.1002/vms3.70398

**Published:** 2025-05-19

**Authors:** Emiliano Ariel Videla, Jorge Martin Caliva, Romina Paula Andrea Picco, Raúl Héctor Marín, Agustin Luna, Franco Nicolas Nazar

**Affiliations:** ^1^ Universidad Nacional de Córdoba (UNC) Facultad de Ciencias Exactas Físicas y Naturales Instituto de Ciencia y Tecnología de los Alimentos (ICTA) Córdoba Argentina; ^2^ Consejo Nacional de Investigaciones Científicas y Técnicas (CONICET) Instituto de Investigaciones Biológicas y Tecnológicas (IIBYT, CONICET‐UNC) Córdoba Argentina; ^3^ Microfluidics Cluster UPV/EHU, Analytical Microsystems & Materials for Lab‐on‐a‐Chip (AMMa‐LOAC) Group University of the Basque Country UPV/EHU Vitoria‐Gasteiz Spain

**Keywords:** immunity, natural feed additives, poultry, supplementation, welfare

## Abstract

**Background:**

Poultry housing includes unavoidable stressors that impair birds’ welfare and health. Global policies are banning antimicrobial growth promoters mainly due to antimicrobial resistance. Antioxidants modulate immunity by reducing oxidative stress, which impairs immune function. Thymol (THY), tocopherol (TOC) and ascorbyl palmitate (AP) have been studied for their antioxidant, antimicrobial and anxiety/fear‐reducing properties. However, their effects on immune function and stress responses in broilers require further investigation.

**Objectives:**

This study assesses whether dietary THY or a TOC and AP mix modulates immune and chronic stress‐related responses in broilers.

**Methods:**

Cobb‐500 chicks (*n* = 960) were assigned to one of 6 dietary treatments: (1) Basal (control), (2) Promotor (Basal + flavomycin), (3) BHT (Basal + butylated hydroxytoluene), (4) Promotor‐BHT (Basal + flavomycin + BHT), (5) THY (Basal + thymol) and (6) TOC‐AP (Basal + tocopherol + AP). Immune and stress parameters were evaluated, including inflammatory response to phytohemagglutinin‐P (PHA‐P), antibody production against sheep red blood cells (SRBC) and heterophil‐to‐lymphocyte (H/L) ratio.

**Results:**

Supplementation did not affect the PHA‐P inflammatory response (*p* = 0.72) but influenced SRBC antibody production and H/L ratio (*p* < 0.003). Broilers supplemented with THY or TOC‐AP exhibited significantly lower antibody responses, potentially avoiding an energy‐demanding acquired immune activation. Additionally, both groups showed significantly lower H/L ratios, suggesting that these supplements may help mitigate physiological stress induced by routine husbandry practices.

**Conclusion:**

These findings provide evidence that THY and TOC‐AP may serve as natural alternative to synthetic additives for improving welfare and mitigating stress‐induced immune imbalance under commercial rearing conditions.

## Graphical abstract

Poultry housing stress impairs welfare and health, whereas antimicrobial growth promoters are being banned due to resistance concerns. This study evaluates thymol (THY) and a tocopherol–ascorbyl palmitate mix (TOC‐AP) supplementation in broilers, showing reduced antibody responses and heterophil‐to‐lymphocyte ratios, suggesting potential benefits for mitigating stress‐induced immune imbalances and improving welfare.

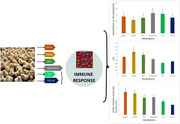

## 1 Introduction

Poultry production inherently subjects birds to stressors such as transportation, handling, vaccination, restricted feeding programmes, overcrowding and social structure changes during rearing stages. These stressors can impair performance and welfare, as restoring the homeostasis is energetically demanding (Scanes 2016; Lu et al. 2021; Romero; et al. 2009). A competent immune system is vital for protecting poultry against pathogens and comprises two major components: an innate response that acts rapidly but non‐specifically, and an acquired response involving specialised cells that develops more slowly yet provides targeted defence (Lee 2006; Buehler et al. 2010; Valdebenito et al. 2018). When immune function is compromised, disease susceptibility increases, diverting resources from production to defence mechanisms (Rauw 2012).

Historically, synthetic antibiotics have been widely used for therapeutic purposes and as growth promoters in poultry (Yadav et al. 2016). However, concerns over potential toxicity and the emergence of antimicrobial resistance have prompted regulatory restrictions and bans in various regions (Nechitailo et al. 2024; SENASA 2024). This shift has fuelled interest in alternative feed additives, particularly phytogenic compounds and antioxidants, which have shown to support immune function by reducing oxidative stress and mitigating excessive immune activation (Abdelli et al. 2021; Kiran Kumar et al. 2022). These compounds also offer potential benefits for poultry health, welfare and productivity (Luna et al. 2010; Abdelli et al. 2021).

Thymol (THY), a primary component of essential oils from oregano (*Origanum vulgare*) and thyme (*Thymus vulgaris*), has been reported to exhibit antioxidant, antimicrobial and stress‐modulating properties, potentially through GABAergic (gamma‐aminobutyric acid (GABA) system) pathways (Lábaque et al. 2013; Delgado‐Marín et al. 2017; Martemucci et al. 2022). On the other hand, alpha‐tocopherol (TOC), a form of vitamin E, is a lipid–soluble antioxidant that protects cell membranes from oxidative damage, whereas ascorbyl palmitate (AP), a lipid–soluble derivative of vitamin C, works synergistically with TOC to enhance antioxidant capacity and modulate immune responses (Jampilek and Kralova 2020; Herrero‐Encinas et al. 2023). Although the individual effects of these compounds have been studied, there is limited research directly comparing their effects in separate treatment groups under the same experimental conditions.

This study aims to evaluate and compare the effects of THY, TOC‐AP, flavomycin and butylated hydroxytoluene (BHT) on immune function and stress‐related indicators in broilers. THY, TOC and AP were selected for their antioxidant and immunomodulatory properties, whereas BHT serves as a synthetic antioxidant control and flavomycin as an antibiotic growth promoter for comparison. However, there is a lack of studies that have directly compared these natural and synthetic additives within the same experimental framework, specifically assessing their effects on key immune and stress‐related indicators in broilers. Herein, we assessed (1) pro‐inflammatory status, measured by the inflammatory response to phytohemagglutinin‐P (PHA‐P), a widely used test of cell‐mediated immune function, primarily driven by T‐cell activity; (2) the acquired immune response—assessed through antibody production against sheep red blood cells (SRBC), a standard method for evaluating humoral immunity in poultry and (3) chronic stress, evaluated using the heterophil‐to‐lymphocyte (H/L) ratio, an established physiological indicator of prolonged stress exposure and corticosterone‐mediated immune shifts in poultry. Furthermore, this study uses molar‐equivalent dosing for compound antioxidant comparisons, addressing a common limitation in previous studies that relied on weight‐equivalent doses. Weight‐based comparisons do not account for differences in molecular weight or reaction stoichiometry, leading to inaccurate assessments of antioxidant efficacy. Because these compounds show antioxidant properties that neutralise radicals through hydrogen donation from their hydroxyl groups, molar equivalence provides a more precise basis for comparing their biological and physiological effects.

To isolate the effects of the additives, newly hatched male broiler chicks of uniform body weight were randomly assigned to one of five dietary groups: a basal control diet or diets supplemented with THY, TOC‐AP, flavomycin or BHT. No other combinations were tested, ensuring that the effects of those compounds could be independently evaluated. Over a 7‐week period, performance parameters (body weight, feed intake and feed conversion ratio) were monitored, alongside periodic assessments of immune and stress responses.

By integrating a direct comparison of natural and synthetic additives with a rigorous molar‐based dosing approach, this study provides novel insights into the physiological effects of alternative feed additives under commercial poultry production conditions.

## 2 Materials and Methods

### 2.1 Animals and Husbandry

This study was conducted on a commercial farm following standard care and husbandry protocols recommended by the Argentine National Service for Agri‐Food Health and Quality (SENASA). The study complied with applicable Argentine laws and adhered to the guidelines of the Argentine Association for Science and Technology of Laboratory Animals (Avalos and Belchior 2015).

One‐day‐old Cobb‐500 male broiler chicks with similar weight (43.0 ± 1.0) g were obtained from a local hatchery (INDACOR S.A.) and located in a commercial farm operated by the company. Upon arrival, birds were identified (leg banded for 2 weeks and wing banded after that) and randomly housed in groups of 40 individuals in one of 24 pens measuring 2.4 × 1.2 m^2^. Water and feed were provided ad libitum. Dietary supplements were dissolved in soybean oil, sprayed onto the basal diet and mixed thoroughly before extrusion to ensure homogeneity (Luna et al. 2010).

### 2.2 Treatment Procedures

All birds within each pen were randomly assigned to one of six dietary treatments. There were four replicates (pens) of each dietary treatment. Each replica initially consisted of 40 birds. Dietary treatments were characterized as follows: (1) Basal (no supplements added), (2) Promotor (Basal + 6.26 µmol flavomycin/kg feed), (3) BHT (Basal + 1.33 mmol of BHT/kg feed), (4) Promotor‐BHT (Basal + a mix of 6.26 µmol flavomycin/kg feed and 1.33 mmol of BHT/kg feed), (5) THY (Basal + 1.33 mmoles of THY/kg feed) and (6) TOC‐AP (Basal + 0.665 mmoles of TOC + 0.665 mmoles of AP/kg feed). The Basal, Promotor (flavomycin 8%, Hugestone Enterprise Co., China), BHT (FlukaAG, Buchs SG, Switzerland) and Promotor‐BHT, were used as controls. THY (SAFCR, ≥99%, FCC, USA) and TOC‐AP (GRINDOX 497 DuPONT, Danisco Argentina) were considered the experimental groups. When birds were 21 days (d) old, stocking density in each pen was randomly reduced to 32 birds per pen. Dosages were selected on the basis of prior research and commercial farm practices (Lábaque et al. 2013; Bodoira et al. 2017; Delgado‐Marín et al. 2017; Herrero‐Encinas et al. 2023). All supplement concentrations were standardized to 1.33 mmol/kg, corresponding to 200 mg/kg of THY and 293 mg/kg of BHT. The selected BHT dose falls within the range commonly used in poultry production, ensuring its relevance for industry comparisons. THY was included at a molar‐equivalent dose to BHT, as both compounds react with free radicals through their hydroxyl groups in a 1:1 molar ratio (also applicable to TOC and AP). This standardization ensures that dietary trials evaluating antioxidant activity are directly comparable. Flavomycin was included at commercial doses.

The feeding regimen consisted of a starter (1–10 days), grower (11–26 days) and finisher (27–42 days) diet. The ingredients and chemical composition of the basal diets are detailed in Table 1. Birds were raised following a standard husbandry programme for slaughter weight between 2.5 and 3.0 kg (Cobb‐vantress; Broiler Management Guide 2021).

**TABLE 1:** Feed composition of each basal experimental diet where dietary supplements were added.

Composition of basal diets (kg %)			
Compound	Starter	Grower	Finisher
**Maize**	47.6	51.2	61.67
**Soybean pellets**	34.3	28.6	17.55
**Soybean**	8.7	13.50	13.17
**Animal meal**	4.75	1.50	3.25
**Crude soy oil**	2.5	2.50	1.95
**DL methionine 88% (liq.)**	0.55	0.47	0.37
**Salt**	0.32	0.32	0.3
**Emulsifier**	0.25	0.25	0.03
**l‐Lysine HCL**	0.22	0.24	0.22
**Bisodium/Bisulfate butyrate**	0.2	0.3	0.3
**Mineral Premix** ^a^	0.15	0.15	0.1
**Vitamin Premix** ^b^	0.15	0.12	0.1
**l‐Threonine**	0.12	0.1	0.07
**Acidity reg. (sodium lactate)**	0.1	0.15	0.2
**Choline chloride**	0.07	0.07	0.07
**Limestone powder**	—	0.52	0.6
**Crude protein, %**	22.2	19.9	18.2
**ME, kcal/kg**	12.5	12.6	12.8
**Calcium, %**	8.90	8.70	7.70
**Phosphorous (total)**	6.8	6.0	6.0

^a^ Mineral premix provided the following amounts per kilogram of diet: Mn, 11.0%; Zn, 11.0%; Fe, 6.0%; I, 2.0 ppm; Mg, 2.68%; Se, 600 ppm.

^b^ Vitamin premix provided the following amounts per kilogram of diet: vitamin D3, 200 IU; vitamin A, 1.500 IU; vitamin E, 101 IU; niacin, 35 mg; d‐pantothenic acid, 14 mg; riboflavin, 4.5 mg; pyridoxine, 3.5 mg; menadione, 2 mg; folic acid, 0.55 mg; thiamine, 1.8 mg.

### 2.3 Sampling Procedures

On Day 35 of age (1 week before slaughtering), blood samples were taken from the left brachial vein of 10 broilers from each of the 24 pens (40 birds in total from each dietary treatment). Blood withdrawal was done with ethylenediaminetetraacetic acid to avoid blood coagulation; anaesthesia was not used in line with guidance in the National Centre for the Replacement Refinement and Reduction of Animals in Research (www.nc3rs.org.uk/general‐principles). The sampling procedure was randomly performed between treatments. One blood drop was used for blood smears, and the rest was centrifuged at 2500 *g* for 15 min to obtain plasma, pellets were discarded. Plasma samples were stored at −80°C and then used for microagglutination assay to determine humoral response against SRBC.

### 2.4 Immunological Determinations

To assess the inflammatory response, the day before blood samples were taken, the peripheral zone of the right‐wing web was measured with a digital calliper and then intradermally injected with 0.1 mL of PHA‐P (Sigma Chemical, St Louis, USA) solution in phosphate saline buffer (pH = 7, 1 mg/mL) (Vinkler et al. 2010). Twenty‐four hours later the same region previously injected was measured to determine the percentage of inflammation, calculated using the following formulae: percentage of inflammation = [(inflammation previous 24 h)/(inflammation post 24 h)] × 100.

For the microagglutination assay, a 30 µL aliquot of plasma was used as a pure sample. Another 30 µL aliquot of plasma was serially diluted in 30 µL of PBS across 10 wells of a 96 U well plate. Then, 30 µL of a 2% solution of SRBC were added to each well, except for the blanks. The 11 and the 12 wells were used as blank (serum diluted with PBS only) and negative controls (PBS with SRBC), respectively. Plates were incubated for 45 min at 40°C. Antibody titres were reported as the Log_2_ of the highest dilution yielding significant agglutination.

Blood smears were stained with May–Grünwald Giemsa. Leukocyte differentiation was manually performed by a trained observer using a light optical microscope (1000× magnification). A total of 100 white cells per smear were counted and differentiated in heterophils, basophils, eosinophils, monocytes and lymphocytes. The heterophil to lymphocyte ratio was calculated for each individual. H/L ratio was calculated using the following formulae: H/L ratio = (number of heterophils)/(number of lymphocytes) (Nazar et al. 2018).

### 2.5 Statistical Analysis

General linear and mixed models were used to compare differences between groups on each response variable. According to the data distribution, the percentage of inflammation, titres against SRBC and H/L ratio variables were square root, square root and log10 transformed, respectively, in order to reach homogeneity. Dietary treatment was defined as a fixed effect, and DGC test was performed for post hoc analysis. A *p* value < 0.05 was considered to represent significant differences. Statistical analyses were performed through an ‘R’ (Ihaka and Gentleman 1996, version 4.4.1) interface and InfoStat (version 2016; Di Rienzo et al. 2016).

## 3 Results

A summary of the results, including mean values and statistical comparisons for all response variables, is presented in Table 2.

**TABLE 2:** Effects of dietary treatments on the percentage of inflammation, titres against sheep red blood cells and heterophil to lymphocyte ratio in male broiler chickens.

	Treatment (mean ± SEM)						
Response variable	Basal	PROMOTOR	BHT	PROMOTOR‐BHT	THY	TOC‐AP	*p* value
Percentage of inflammation	14.71 ± 2.65	12.99 ± 2.13	13.24 ± 2.22	18.27 ± 3.48	16.07 ± 1.98	14.42 ± 2.00	0.72
Titres against sheep red blood cells (Log_2_)	4.46 ± 0.50^a^	4.26 ± 0.51^a^	3.55 ± 0.41^a^	3.54 ± 0.50^a^	2.60 ± 0.44^b^	2.29 ± 0.50^b^	0.001
Heterophil/Lymphocyte ratio	1.00 ± 0.06^a^	1.31 ± 0.35^a^	1.06 ± 0.10^a^	0.88 ± 0.06^a^	0.63 ± 0.04^b^	0.73 ± 0.06^b^	0.003

*Note*: Basal = no feed supplements added; PROMOTOR = Basal + 6.26 µmoles flavomycin/kg feed; BHT = Basal + 1.33 mmoles of buthylated hidroxytoluene (BHT)/kg feed; Promotor‐BHT = Basal + a mix of 6.26 µmoles flavomycin/kg feed and 1.33 mmoles of BHT/kg feed; THY = Basal + 1.33 mmoles of thymol/kg feed; TOC‐AP = Basal + 0.67 mmoles of tocopherol + 0.67 mmoles of ascorbyl palmitate/kg feed. Means within a row with no common superscripts (a and b) differ significantly at *p* < 0.05.

Abbreviation: SEM, standard error of the mean.

Dietary supplementation had no significant effect on the percentage of inflammation induced by PHA‐P injection (*F*₅,₁₇₂ = 0.57; *p* = 0.72). Mean inflammation responses ranged from 12.99% ± 2.13% (Promotor) to 18.27% ± 3.48% (Promotor‐BHT), with no significant differences among groups. Therefore, neither THY, TOC‐AP, BHT nor flavomycin altered the local pro‐inflammatory response compared to the control group.

A significant effect of dietary supplementation was observed for SRBC antibody titres (*F*₅,₁₆₉ = 4.87; *p* < 0.001). Post hoc analyses revealed that broilers supplemented with THY (2.60 ± 0.44) and TOC‐AP (2.29 ± 0.50) exhibited significantly lower antibody titres compared to the Basal diet (4.46 ± 0.50), the Promotor flavomycin (4.26 ± 0.51), BHT (3.55 ± 0.41) and Promotor‐BHT (3.54 ± 0.50) groups (*p* < 0.05). No significant differences were observed between the THY and TOC‐AP groups (*p* = 0.64), showing that both treatments similarly modulated the humoral immune response.

For the H/L ratio, a key indicator of chronic stress, dietary supplementation significantly affected this parameter (*F*₅,₁₁₈ = 3.87; *p* < 0.003). Birds receiving THY (0.63 ± 0.04) and TOC‐AP (0.73 ± 0.06) had significantly lower H/L ratios than those in the Basal (1.00 ± 0.06), Promotor (1.31 ± 0.35), BHT (1.06 ± 0.10) and Promotor‐BHT (0.88 ± 0.06) groups (*p* < 0.05). These findings show that THY and TOC‐AP supplementation are associated with reduced physiological stress, as indicated by the lower H/L ratios.

## 4 Discussion

This study evaluated the effects of dietary supplementation with THY and a combination of TOC and AP on immune response and stress indicators in broilers, comparing them to two commercially used supplements, flavomycin and BHT. Immune function was assessed through PHA‐P‐induced inflammation and antibody production against SRBC, whereas the H/L ratio was used as an indicator of chronic stress.

No significant differences were observed in PHA‐P‐induced inflammation across treatments, suggesting that neither THY nor TOC‐AP exerted strong pro‐ or anti‐inflammatory effects under the study conditions. The PHA‐P assay is widely used to evaluate in vivo pro‐inflammatory capacity, as it involves an integrated immune response from macrophages, heterophils, lymphocytes, cytokines and reactive oxygen species (Koutsos and Klasing 2001; Vinkler et al. 2010). An exaggerated inflammatory response can lead to tissue damage, whereas an insufficient response may weaken the ability to fight infections (Davison 2022). The absence of significant differences indicates that THY and TOC‐AP did not disrupt immune homeostasis by excessively stimulating or suppressing inflammation, suggesting that their primary physiological effects may be exerted through other immune pathways, such as modulation of the acquired immune response or oxidative stress regulation (Klasing 2007). Additionally, the inflammatory response is highly context‐dependent, influenced by factors such as dosage, diet composition and the physiological status of the birds (Broom and Kogut 2018; Abdelli et al. 2021). The prior research has reported both anti‐inflammatory (Li et al. 2023) and pro‐inflammatory (Hashemipour et al. 2013) effects of THY, reinforcing the idea that its immunomodulatory effects are dependent on external conditions. In this study, the stable inflammatory response across treatments may reflect a beneficial role of THY and TOC‐AP in maintaining immune balance without triggering unnecessary immune activation, which could be advantageous in preventing excessive energy allocation to inflammatory processes while maintaining adequate immune defence (Broom and Kogut 2018).

Broilers supplemented with THY and TOC‐AP exhibited lower SRBC antibody titres compared to controls (fed the basal diet, flavomycin or BHT). Although this finding aligns with the previous research on THY supplementation (Nazar et al. 2018), it contrasts with studies reporting enhanced antibody responses following TOC supplementation (Kakhki et al. 2016). Several mechanisms could underlie this result. THY, TOC and AP are highly lipophilic compounds (TOC more than AP and both more lipophilic than THY) that interact with biological membranes, potentially altering immune cell signalling (Mohd Zaffarin et al. 2020; Gholami‐Ahangaran et al. 2022). Additionally, THY has antimicrobial properties that may influence antigen exposure and immune activation (Escobar et al. 2020). Given these factors, a lower antibody response does not necessarily indicate immunosuppression but may reflect a shift in immune resource allocation or a context‐dependent modulation of immune function. A possible explanation is that a reduced antigenic challenge led to a lower acquired immune response. Alternatively, immune modulation may have occurred as a trade‐off between innate and adaptive immunity, a phenomenon reported in previous poultry studies (Buehler et al. 2010). Further research is required to determine whether this effect is associated with improved immune efficiency or context‐dependent regulatory shifts in immune function under different production conditions or when exposed to pathogen‐challenging conditions.

Broilers receiving THY and TOC‐AP exhibited the lowest H/L ratios, suggesting a reduced physiological response to chronic stressors. The H/L ratio is a well‐established indicator of circulating stress mediators, where higher values are associated with increased corticosterone levels and prolonged stress exposure (Scanes 2016; Nazar et al. 2018, 2019). Several factors may contribute to this finding. THY has been reported to modulate stress responses in poultry, potentially through interactions with neurotransmitter systems (Lábaque et al. 2013; Videla et al. 2020). Similarly, ascorbic acid derivatives, such as AP, have been implicated in catecholamine metabolism, which could influence neuroendocrine pathways involved in stress regulation (Delgado‐Marín et al. 2017). However, although these mechanisms are plausible, our study did not directly measure neurobiological markers, and these interpretations should be made with caution. Thus, our findings should be understood within the context of immune modulation and stress physiology rather than energy conservation or neurotransmission effects. Further research should explore these potential interactions by assessing specific neuroendocrine and metabolic markers.

Although this study focused on a limited number of immune‐ and stress‐related markers, the selected parameters—PHA‐P‐induced inflammation, SRBC antibody production and the H/L ratio—are widely recognized as robust indicators of immune modulation and stress responses in poultry. These markers align with the previous research in avian immunology and welfare assessment (Vinkler et al. 2010; Koutsos and Klasing 2001; Scanes 2016; Nazar and Marin 2011; Nazar et al. 2020, 2018; Hoffman et al. 2020; Lee 2006). Although additional data on cytokine profiles or oxidative stress markers could provide further insights, the measured variables offer a meaningful and practical evaluation of dietary interventions under commercial poultry conditions. Future studies could expand on these findings by incorporating broader immunological and metabolic assessments.

## 5 Conclusion

Feed supplementation with THY and TOC‐AP was associated with lower SRBC antibody production and reduced H/L ratios in broilers compared to the basal, flavomycin and BHT groups, without significantly altering inflammatory responses. The lower antibody response does not necessarily imply immunosuppression but may reflect context‐dependent immune modulation. The reduction in the H/L ratio suggests a potential role in mitigating stress‐related physiological alterations, which could have welfare benefits under commercial poultry production conditions. These effects were not observed with BHT or flavomycin, highlighting the potential of THY and TOC‐AP as natural alternatives to conventional dietary supplements. Future studies should explore the long‐term effects of these compounds on immune competence and stress resilience, as well as their potential interactions with other nutritional and environmental factors in broiler production.

## Author Contributions

**Emiliano Ariel Videla**: data curation, investigation, writing – original draft preparation. **Jorge Martin Caliva**: data curation, investigation, formal analysis. **Romina Paula Andrea Picco**: writing – original draft preparation and formatting. **Raúl Héctor Marín**: conceptualization, resources, funding acquisition, writing – review and editing. **Agustin Luna**: conceptualization, supervision, visualization, project administration, validation, review and editing final manuscript. **Franco Nicolas Nazar**: conceptualization, writing – review and editing final manuscript, methodology, supervision.

## Ethics Statement

The study complies with applicable Argentinean laws, with the local Argentinean Association for Science and Technology Laboratory Animals (AACyTAL Bulletins number 15 and 16, 2001). Birds were reared under commercial conditions following Cobb guidelines and the Argentinian SENASA (Servicio Nacional de Sanidad y Calidad Agroalimentaria) regulations.

## Conflicts of Interest

The authors declare no conflicts of interest.

### Peer Review

The peer review history for this article is available at https://www.webofscience.com/api/gateway/wos/peer‐review/10.1002/vms3.70398.

## Data Availability

The data that support the findings of this study are openly available in the CONICET repository at [https://ri.conicet.gov.ar/handle/11336/241615].
